# Description of national antibiotic prescribing rates in U.S. long-term care facilities, 2013–2021

**DOI:** 10.1017/ash.2024.457

**Published:** 2024-11-21

**Authors:** Katryna A. Gouin, Stephen Creasy, Mary Beckerson, Martha Wdowicki, Lauri A. Hicks, Sarah Kabbani

**Affiliations:** 1 Division of Healthcare Quality Promotion, National Center for Emerging and Zoonotic Infectious Diseases, U.S. Centers for Disease Control and Prevention, Atlanta, GA, USA; 2 PharMerica, A BrightSpring Health Services Company, Louisville, KY, USA

## Abstract

Long-term care pharmacy dispensing data from 2013 to 2021 were used to characterize antibiotic prescribing data in U.S. long-term care facilities. Overall antibiotic prescribing rates decreased from 2013 to 2021, mostly due to decreases in fluoroquinolones and macrolides. Tracking antibiotic use in long-term care settings can help identify opportunities for optimizing prescribing practices.

## Introduction

Residents in long-term care (LTC) facilities are at increased risk of adverse events from antibiotic prescribing due to multiple comorbidities, increased likelihood of drug interactions, and risk of infection with antibiotic-resistant organisms and *Clostridioides difficile*.^1,2^ Studies estimate that more than half of LTC residents will be prescribed an antibiotic in a year, and up to 75% of prescriptions are inappropriate.^3^ Actionable and timely data are needed to describe antibiotic use in LTC facilities, both at the local and national levels. The objective of this analysis was to use antibiotic prescribing data elements captured within pharmacy dispense data to describe changes in antibiotic use measures in U.S. LTC facilities from 2013 to 2021.

## Methods

We conducted a retrospective descriptive analysis of antibiotic dispense data for 2013–2021 provided by LTC pharmacy PharMerica, a BrightSpring Health Services Company. We included systemic (oral and parenteral) antibiotics identified by American Hospital Formulary Service codes 0812**, 0836**, and 0892** and excluded local and topical antibiotics. Dispense-level antibiotic data consisted of antibiotic class and agent, route of administration, date of prescription dispense, prescription days supplied, and coded resident and facility identifiers. The prescription end date was calculated from the prescription days supply. The pharmacy provided monthly census data containing resident days contributed by each resident in a specific LTC facility by assessing the admit and discharge dates of residents in relation to the month of service of their prescriptions. We included LTC facilities that reported ≥1 anti-infective dispense for ≥4 months with census data for 12 months.

There may be multiple antibiotic prescriptions dispensed from the LTC pharmacy to the same resident that make up one antibiotic treatment course.^4^ Thus, we combined multiple dispenses of the same drug to the same resident if the subsequent dispense was within 3 days of the preceding end date to define one antibiotic course. The antibiotic course duration was calculated as the difference between the calculated end date and the initial prescription dispensing date. Antibiotic Days of Therapy (DOT) were calculated as the sum of all course durations for specific agents and overall. Overall antibiotic use was reported as the percent of residents receiving an antibiotic per year, antibiotic courses per 1,000 resident-days, and antibiotic DOT per 1,000 resident-days overall and by route of administration, antibiotic class, and agent. We reported median course duration with interquartile range (IQR). We also described courses as the percent of the following durations: 1–7 days, 8–14 days, 15–42 days, and >42 days. To compare antibiotic course incidence rates in 2013 and 2021, we estimated prevalence rate ratios (pRRs) and 95% confidence intervals (CIs) using Poisson models with a log link. We used the year 2013 as the reference to estimate changes overall and by route, antibiotic class, and agent. We also reported pRRs comparing 2013 to 2019 and 2019 to 2021 to measure changes in antibiotic prescribing rates prior to and during the COVID-19 pandemic. This analysis was conducted using SAS 9.4 (SAS Institute, Cary, NC).

## Results

**Figure 1.**
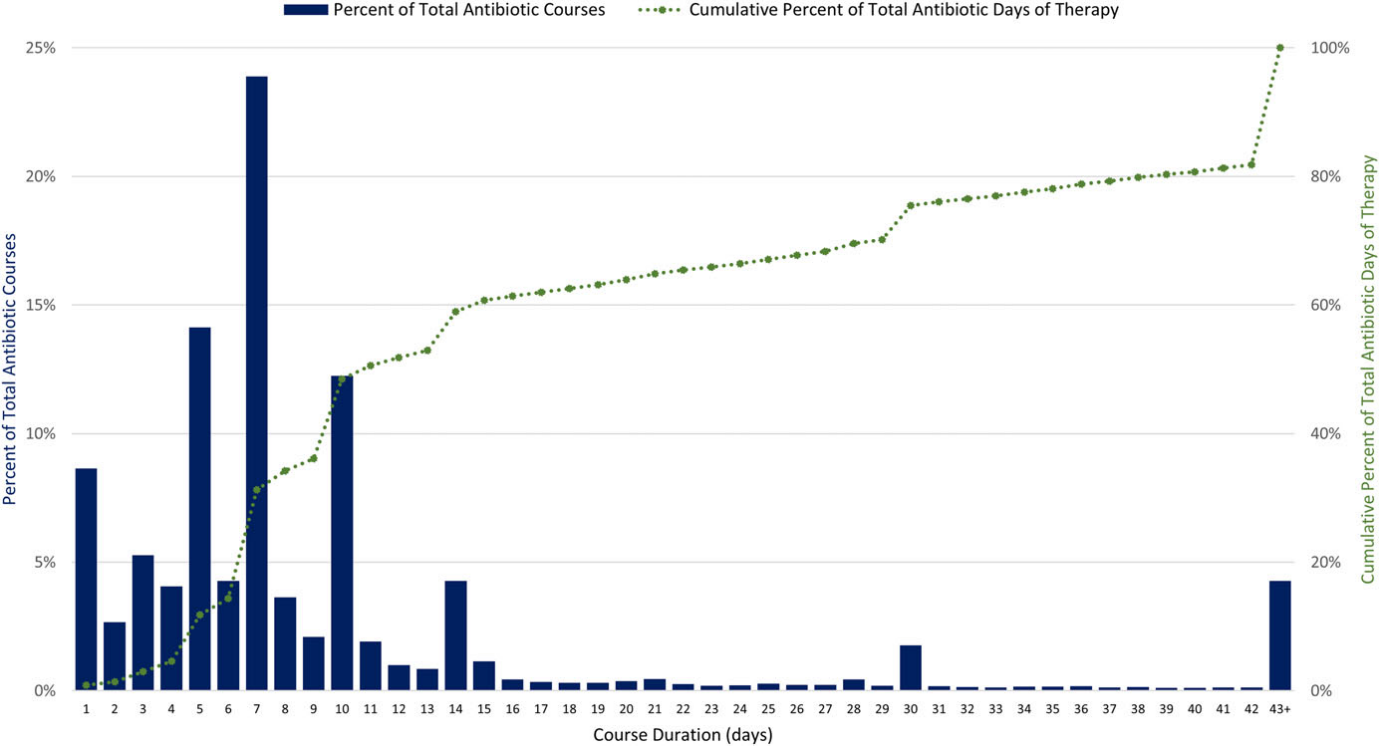
Distribution of antibiotic course duration and cumulative percent of total antibiotic days of therapy for 296,572 antibiotic courses in 1,378 U.S. long-term care facilities, 2021. Source: PharMercia, a BrightSpring Health Services Company.

We analyzed data on antibiotics dispensed in 1,900 unique U.S. LTC facilities from 2013 to 2021, representing approximately 12% of the number of LTC facilities nationwide. Antibiotic dispenses in 2021 were combined into 296,572 antibiotic treatment courses (50% of dispenses were combined) (Figure S1). In 2021, total DOT was 2,935,191 (Table S1). Of all antibiotic courses prescribed in 2021, 63% were 1–7 days, 26% were 8–14 days, and 9% were 15–42 days in length (Figure 1). Long-term antibiotic courses, defined as those longer than 42 days, occurred in 1,150 LTC facilities (83%) and contributed to 4% of all courses and 18% of overall DOT. The most common agents prescribed for longer than 42 days were nitrofurantoin, sulfamethoxazole/trimethoprim, and doxycycline.

From 2013 to 2021, the percentage of residents receiving an antibiotic decreased from 51% to 44%. Total antibiotic use rates in antibiotic courses/1,000 resident-days decreased by 8% (pRR 0.92, CI 0.91–0.93) from 2013 to 2021; there was a 3% decrease from 2013 to 2019 and a 5% decrease from 2019 to 2021 (Table 1). Total antibiotic use rates in DOTs/1,000 resident-days decreased by 8% from 2013 to 2021, with an 8% decrease occurring from 2013 to 2019 and no change from 2019 to 2021 (Figure S1). The median antibiotic course duration was 7 days (IQR: 5–10) in 2013 and 2021.

**Table 1.** Changes in antibiotic use rates in long–term care facilities, 2013 versus 2021–United States

	Antibiotic courses per 1000 resident–days, incidence			
	2013	2021	pRR 2021 v 2013^a^ (95% CI)	Percent change
Overall	9.22	8.47	0.92 (0.91–0.93)	–8%
**Route**				
Oral	7.75	7.00	0.90 (0.90–0.91)	–10%
Intravenous (IV)	1.46	1.47	1.00 (0.99–1.02)	0%
**Antibiotic class**				
Cephalosporins	1.85	2.25	1.22 (1.20–1.23)	22%
Fluoroquinolones	2.71	1.39	0.51 (0.51–0.52)	–49%
Penicillins	1.03	1.16	1.13 (1.11–1.15)	13%
Antibacterials, misc.^b^	0.62	0.87	1.41 (1.38–1.45)	41%
Tetracyclines	0.53	0.83	1.56 (1.52–1.60)	56%
Sulfonamides	0.87	0.65	0.74 (0.73–0.76)	–26%
Urinary anti–infectives	0.66	0.57	0.86 (0.84–0.89)	–14%
Macrolides	0.66	0.46	0.70 (0.68–0.71)	–30%
Beta-lactams, misc.^c^	0.17	0.25	1.44 (1.37–1.50)	44%
Aminoglycosides	0.11	0.05	0.38 (0.36–0.41)	–62%
**Antibiotic agent**				
Cephalexin	0.76	0.89	1.18 (1.15–1.20)	18%
Doxycycline	0.46	0.78	1.71 (1.66–1.76)	71%
Amoxicillin/potassium clavulanate	0.48	0.72	1.49 (1.45–1.53)	49%
Levofloxacin	1.39	0.69	0.51 (0.50–0.52)	–49%
Ciprofloxacin	1.18	0.68	0.58 (0.56–0.59)	–42%
Sulfamethoxazole/trimethoprim	0.87	0.65	0.74 (0.73–0.76)	–26%
Nitrofurantoin	0.63	0.52	0.82 (0.80–0.84)	–18%
Ceftriaxone	0.55	0.49	0.90 (0.88–0.92)	–10%
Azithromycin	0.59	0.43	0.72 (0.70–0.74)	–28%
Vancomycin	0.25	0.43	1.75 (1.69–1.82)	75%
Cefdinir	0.09	0.30	3.28 (3.09–3.48)	228%

Data Source: PharMerica, a BrightSpring Health Services Company.

CI, confidence interval; pRR, prevalence rate ratio.

a pRRs were derived using a Poisson model with a log link, comparing with year as a categorical variable and the year 2013 as the reference.

b Antibacterials, misc. includes agents rifaximin, linezolid, daptomycin, vancomycin, clindamycin, tedizolid phosphate, bismuth/metronidazole/tetracycline, telavancin.

c Beta–lactams, misc. includes agents ertapenem sodium, meropenem, imipenem, aztreonam, cefoxitin, cefotetan.

Prescribing rates of antibiotic courses from 2013 to 2021 decreased across several antibiotic classes: fluoroquinolones decreased by 49% and macrolides decreased by 30%. Specifically, levofloxacin decreased by 49% and azithromycin decreased by 28%. Concurrently, prescribing rates of tetracyclines increased by 56% and cephalosporins increased by 22%. Doxycycline prescribing increased by 71%, cephalexin increased by 18%, and cefdinir increased by 228% (Table 1).

## Discussion

LTC antibiotic prescribing rates decreased in recent years. Regulatory requirements may have played a role in the reduced prescribing rates. The decline in antibiotic prescribing coincides with the Centers for Medicare & Medicaid Services final rule requiring facilities to have a system to track antibiotic use that went into effect in 2017.^5^ The COVID-19 pandemic also greatly affected LTC settings; we reported an overall 8% decrease in antibiotic prescribing rates from 2013 to 2021, but the steepest decline (–5%) occurred between 2019 and 2021. These decreases are likely due to changes in prescribing practices and resident population during the COVID-19 pandemic.^6^

This national assessment highlights potential opportunities to improve treatment duration, as the median course duration of 7 days did not change during the 9-year period. Increasing evidence supports shorter duration of treatment for most infections, and every day of additional antibiotic therapy is associated with increased risk of adverse events.^7,8^ Also, prolonged antibiotic durations contribute 18% of total DOT and provide another opportunity for evaluation of appropriateness of potential prophylaxis or suppressive therapy. Across antibiotic classes, decreases in antibiotic prescribing incidence rates were observed for fluoroquinolones, urinary anti-infectives, and macrolides. Increased awareness of adverse events, including updated Food and Drug Administration’s fluoroquinolone warnings released in 2013, 2016, and 2018, likely contributed to the decline in fluoroquinolones prescribed during the study period.^9^ Increased prescribing of tetracyclines and cephalosporins may be due to clinicians avoiding fluoroquinolones; however, the increased prescribing of cefdinir (228%) should be further evaluated as this agent is not recommended first-line therapy for common infections in older adults. LTC facilities contract with LTC pharmacies for medication management and delivery, and consultant pharmacists can provide stewardship expertise to improve antibiotic selection and duration and support facility staff in tracking antibiotic use data.^10^

This analysis has several limitations. These data may not be generalizable to all LTC facilities. Since the admission date was not available, antibiotic courses started at the facility as well as continuations from hospital-initiated courses were included. We were also unable to stratify rates of antibiotic use by type of resident stay, an important predictor of antibiotic use in LTC settings.^11^ The antibiotic course calculation methodology may overestimate the true number of treatment days (if there are gaps between dispenses of the same drug to the same resident). However, this approach avoids overestimating the number of courses received by a resident. Prescribing indication was also not available limiting the assessment of appropriateness of therapy.

Tracking antimicrobial use in LTC settings can be used to describe baseline prescribing rates, track changes over time, and identify where further evaluation is needed.^12^ LTC pharmacy dispensing data can be used to calculate antibiotic use measures at a facility- and national-level and support stewardship implementation in LTC settings.

## Supporting information

Gouin et al. supplementary material 1 Gouin et al. supplementary material

Gouin et al. supplementary material 2 Gouin et al. supplementary material

Gouin et al. supplementary material 3 Gouin et al. supplementary material

## Supplementary material

The supplementary material for this article can be found at https://doi.org/10.1017/ash.2024.457.
