# Supplementary figures and images for: Description of national antibiotic prescribing rates in U.S. long-term care facilities, 2013–2021
Source: Antimicrob Steward Healthc Epidemiol. 2024 Nov 21;4(1):e209. doi: 10.1017/ash.2024.457 (PMC11626451; doi:10.1017/ash.2024.457)

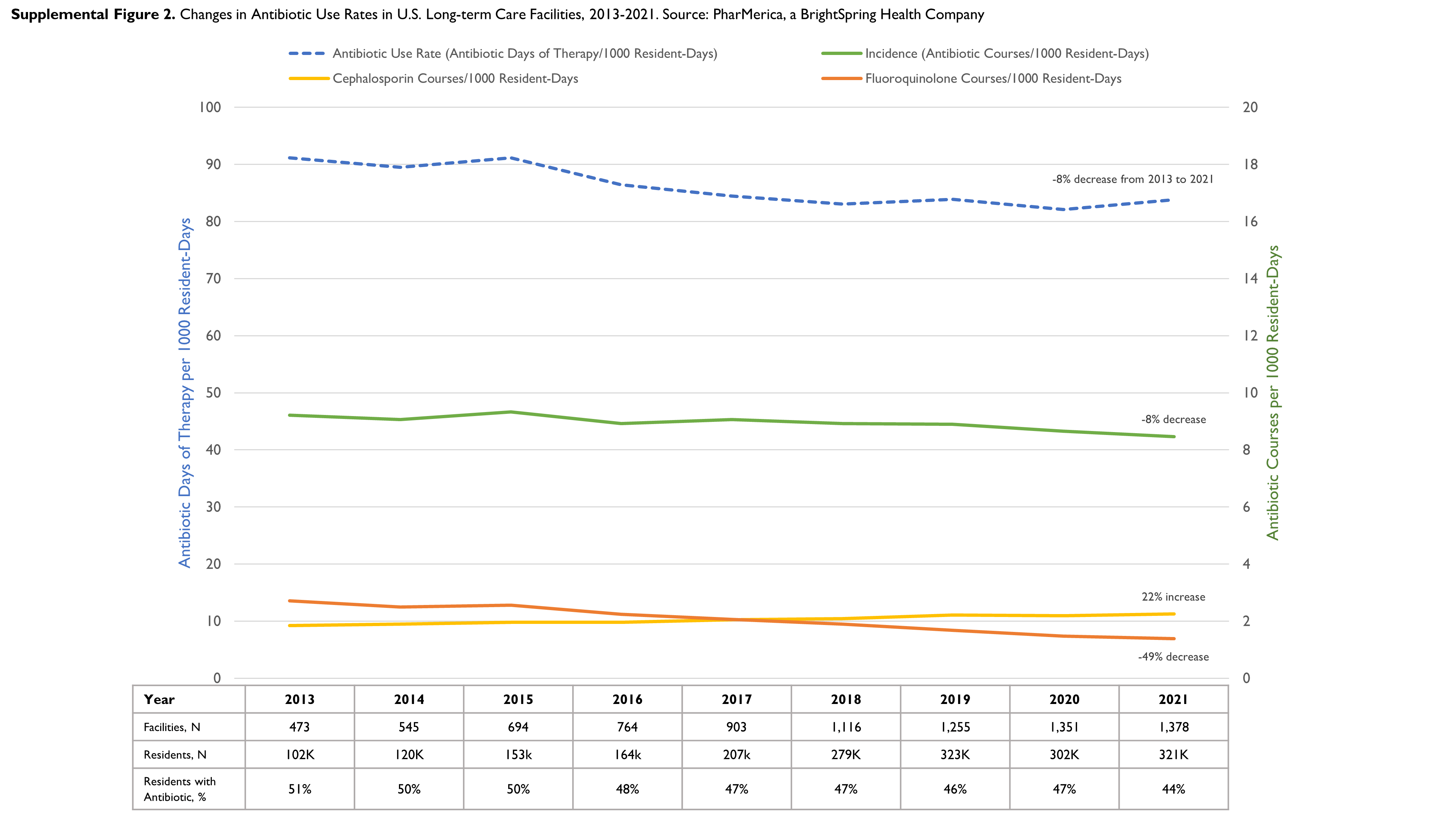

Supplement: Gouin et al. supplementary material 1 — Gouin et al. supplementary material [file S2732494X24004571sup001.tif]
