# Supplementary material for: Description of national antibiotic prescribing rates in U.S. long-term care facilities, 2013–2021
Source: Antimicrob Steward Healthc Epidemiol. 2024 Nov 21;4(1):e209. doi: 10.1017/ash.2024.457 (PMC11626451; doi:10.1017/ash.2024.457)
Supplement: Gouin et al. supplementary material 3 — Gouin et al. supplementary material [file S2732494X24004571sup003.docx]

Supplemental Tables.

Supplemental Table 1. Distribution of antibiotic class and agent by percent of total courses and DOTs in 1,378 long-term care facilities – United States, 2021

|  | **Number of Antibiotic Courses, N** | **%** | **Number of antibiotic courses per 1000 resident-days, Incidence** | **Total Antibiotic Days of Therapy,**  **N** | **%** | **Total antibiotic days of therapy per 1000 resident-days, Utilization** | **Antibiotic course duration** | | |
| --- | --- | --- | --- | --- | --- | --- | --- | --- | --- |
|  |  |  |  |  |  |  | Median | IQR | Mean |
| **Overall** | 296,572 |  | 8.47 | 2,935,191 |  | 83.82 | 7 | 5-10 | 9.9 |
| **Route** | | | | | | | | | |
| Oral | 245,205 | 83% | 7.00 | 2,429,170 | 83% | 69.37 | 7 | 5-10 | 9.9 |
| Intravenous | 51,367 | 17% | 1.47 | 506,021 | 17% | 14.45 | 7 | 3-11 | 9.9 |
| **Antibiotic Class** | | | | | | | | | |
| Cephalosporins | 78,833 | 27% | 2.25 | 678,819 | 23% | 19.39 | 7 | 4-10 | 8.6 |
| Fluoroquinolones | 48,627 | 16% | 1.39 | 373,230 | 13% | 10.66 | 7 | 5-8 | 7.7 |
| Penicillins | 40,503 | 14% | 1.16 | 354,225 | 12% | 10.12 | 7 | 5-10 | 8.7 |
| Antibacterials, misc.^a^ | 30,485 | 10% | 0.87 | 421,951 | 14% | 12.05 | 10 | 6-14 | 13.8 |
| Tetracyclines | 29,137 | 10% | 0.83 | 357,482 | 12% | 10.21 | 7 | 7-10 | 12.3 |
| Sulfonamides | 22,651 | 8% | 0.65 | 265,661 | 9% | 7.59 | 7 | 5-10 | 11.7 |
| Urinary anti-infectives | 20,054 | 7% | 0.57 | 258,569 | 9% | 7.38 | 7 | 5-10 | 12.9 |
| Macrolides | 16,048 | 5% | 0.46 | 122,180 | 4% | 3.49 | 5 | 4-6 | 7.6 |
| Beta-lactams, misc.^b^ | 8,606 | 3% | 0.25 | 83,975 | 3% | 2.40 | 7 | 5-11 | 9.8 |
| Aminoglycosides | 1,628 | 1% | 0.05 | 19,099 | 1% | 0.55 | 7 | 3-10 | 11.7 |
| **Antibiotic Agent** | | | | | | | | | |
| Cephalexin | 31,215 | 11% | 0.89 | 291,845 | 10% | 8.33 | 7 | 5-10 | 9.3 |
| Doxycycline | 27,446 | 9% | 0.78 | 317,214 | 11% | 9.06 | 7 | 7-10 | 11.6 |
| Amoxicillin/potassium clavulanate | 25,074 | 8% | 0.72 | 199,097 | 7% | 5.69 | 7 | 5-10 | 7.9 |
| Levofloxacin | 24,234 | 8% | 0.69 | 175,154 | 6% | 5.00 | 7 | 5-8 | 7.4 |
| Ciprofloxacin | 23,835 | 8% | 0.68 | 187,915 | 6% | 5.37 | 7 | 5-9 | 7.9 |
| Sulfamethoxazole/trimethoprim | 22,641 | 8% | 0.65 | 265,437 | 9% | 7.58 | 7 | 5-10 | 11.7 |
| Nitrofurantoin | 18,175 | 6% | 0.52 | 222,066 | 8% | 6.34 | 7 | 5-10 | 12.2 |
| Ceftriaxone | 17,300 | 6% | 0.49 | 128,287 | 4% | 3.66 | 5 | 2-7 | 7.4 |
| Vancomycin | 15,229 | 5% | 0.43 | 176,607 | 6% | 5.04 | 10 | 5-15 | 12.5 |
| Azithromycin | 14,963 | 5% | 0.43 | 102,079 | 3% | 2.92 | 5 | 4-5 | 6.8 |
| Cefdinir | 10,512 | 4% | 0.30 | 75,211 | 3% | 2.15 | 7 | 5-8 | 7.2 |
| Data Source: PharMerica, a BrightSpring Health Company | | | |  |  |  |  |  |  |

^a^ Antibacterials, misc. includes agents rifaximin, linezolid, daptomycin, vancomycin, clindamycin, tedizolid phosphate, bismuth/metronidazole/tetracycline, telavancin

^b^ Beta-lactams, misc. includes agents ertapenem sodium, meropenem, imipenem, aztreonam, cefoxitin, cefotetan
